# Comparison of Antioxidant Activities of Different Grape Varieties

**DOI:** 10.3390/molecules23102432

**Published:** 2018-09-23

**Authors:** Qing Liu, Guo-Yi Tang, Cai-Ning Zhao, Xiao-Ling Feng, Xiao-Yu Xu, Shi-Yu Cao, Xiao Meng, Sha Li, Ren-You Gan, Hua-Bin Li

**Affiliations:** 1 Guangdong Provincial Key Laboratory of Food, Nutrition and Health, Department of Nutrition, School of Public Health, Sun Yat-sen University, Guangzhou 510080, China; liuq248@mail2.sysu.edu.cn (Q.L.); tanggy5@mail2.sysu.edu.cn (G.-Y.T.); zhaocn@mail2.sysu.edu.cn (C.-N.Z.); xuxy53@mail2.sysu.edu.cn (X.-Y.X); caoshy3@mail2.sysu.edu.cn (S.-Y.C.); mengx7@mail2.sysu.edu.cn (X.M.); 2 Guangzhou No. 2 High School, Guangzhou 510530, China; 18925022122@163.com; 3 School of Chinese Medicine, Li Ka Shing Faculty of Medicine, The University of Hong Kong, Hong Kong, China; 4 Department of Food Science & Technology, School of Agriculture and Biology, Shanghai Jiao Tong University, Shanghai 200240, China; renyougan@sjtu.edu.cn; 5 South China Sea Bioresource Exploitation and Utilization Collaborative Innovation Center, Sun Yat-sen University, Guangzhou 510006, China

**Keywords:** grape, fruit, antioxidant activity, free radical-scavenging ability, phenolics, flavonoid

## Abstract

Grapes are widely consumed in the world, and different grape varieties could exhibit distinctly different antioxidant activities. In this study, the free radical-scavenging and antioxidant activities of lipophilic, hydrophilic, and insoluble-bound fractions from 30 grape varieties were evaluated by ferric-reducing antioxidant powers (FRAP), Trolox equivalent antioxidant capacities (TEAC), total phenolic contents (TPC), and total flavonoid contents (TFC). The results indicated that the 30 grape varieties exhibited diverse FRAP values (1.289–11.767 μmol Fe(II)/g FW), TEAC values (0.339–4.839 μmol Trolox/g FW), TPC values (0.294–1.407 mg GAE/g FW) and TFC values (0.082–0.132 mg QE/g FW). Several grapes, such as Pearl Black Grape (Xinjiang), Summer Black Grape (Shaanxi), Pearl Green Grape (Xinjiang), Seedless Green Grape (Xinjiang), and Seedless Red Grape (Yunnan), exhibited strong free radical-scavenging and antioxidant activities, which could be consumed as good sources of natural antioxidants to prevent several diseases induced by oxidative stress, such as cardiovascular disease and cancer. Furthermore, several antioxidants were identified and quantified, including caffeic acid, catechin gallate, epicatechin, gallic acid, protocatechuic acid and rutin, which could contribute to the antioxidant activities of grapes.

## 1. Introduction

Oxidative stress is involved in a range of chronic diseases, like cancers, diabetes, cardiovascular diseases, Alzheimer’s disease, and Parkinson’s disease [1,2]. Fruits, vegetables, and some other natural products which are rich in antioxidants could reduce oxidative stress in vivo, and might be an effective approach for preventing these diseases [3,4,5,6,7]. However, different kinds of fruits possess various activities [8], contents, and compositions of antioxidants [9]. Even different varieties of a specific fruit species could exhibit different antioxidant capacities and phenolic contents [10,11], and the variation might be very large, depending on many factors, such as cultivars, growing environments, and ripe stages. It has been reported that high consumption of fruits and vegetables, the major dietary sources of antioxidants, could decrease the risk of oxidative stress-related diseases [12]. Therefore, the antioxidant capacities and contents of different varieties are particularly important to estimate the nutritional and medicinal values of fruits.

Grapes are widely consumed all over the world, and have numerous varieties. Some grape varieties have been found to possess notable antioxidant capacities and abundant polyphenols [13], and several antioxidants from grapes have been investigated for their protective effects against many diseases [14,15,16,17]. Some studies compared the antioxidant capacities of wastes (peels and seeds) and products (wine and juice) of a few grape varieties [18,19,20]. Grapes are generally known as important sources of natural antioxidants; however, the differences of antioxidant capacities in different grapes might be very large. Studies on different grapes were very rare, especially studies that include a large number of varieties. Furthermore, different absorption of different phenolic compounds was reported in humans [21], indicating that the phenolic composition of grapes might influence their in vivo antioxidant activities.

This study aimed at determining the free radical-scavenging and antioxidant activities, as well as phenolic contents and compositions, of 30 commonly-consumed grape varieties, and selecting some grapes with high antioxidant capacities and contents. Furthermore, the main phenolic compounds in grape pulps were identified and quantified. This study could be valuable for consumers to select grapes with high nutritional values, and also helpful for producers to cultivate grape varieties with greater health benefits.

## 2. Results and Discussion

### 2.1. Antioxidant Capacities of Grape Pulps

The antioxidant capacities of natural products are usually multifunctional; therefore, more than one assay should be conducted to describe their antioxidant capacities [22]. FRAP and TEAC assays were used in this study. The FRAP assay determines the powers of antioxidants at reducing ferric irons, while the TEAC assay measures the capacities of antioxidants on scavenging ABTS^•+^ free radicals [23].

**Figure 1:**
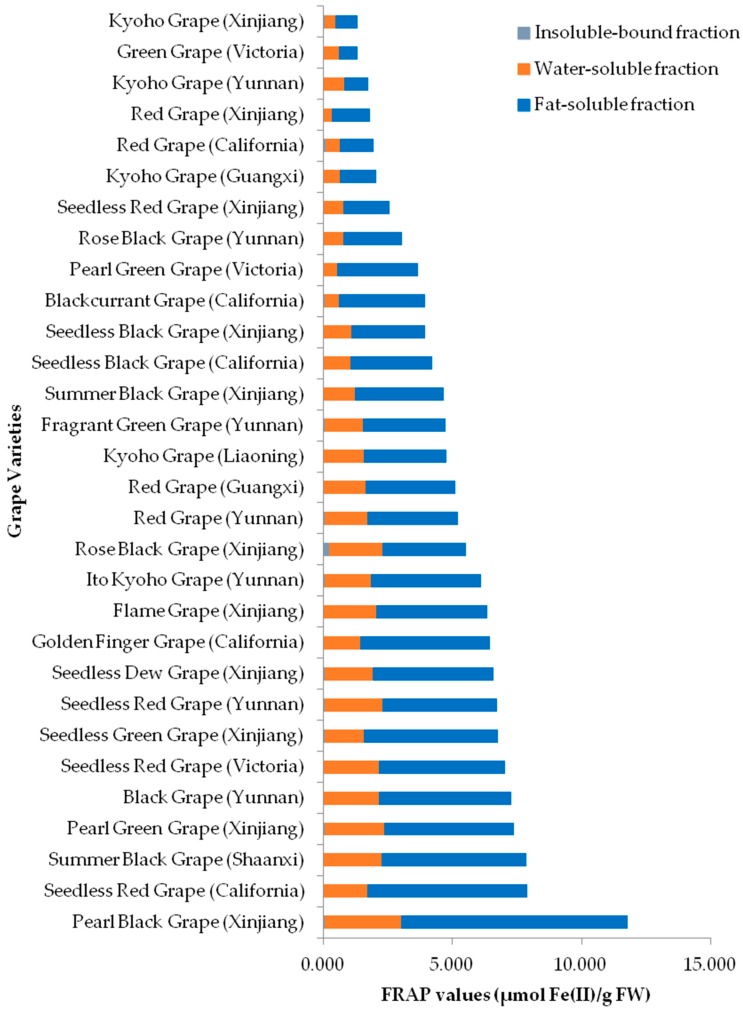
FRAP values of 30 grape pulps.

The FRAP values were in the range of 0.674–8.729 μmol Fe(II)/g fresh weight (FW) for the lipophilic fractions, 0.317–2.967 μmol Fe(II)/g FW for the hydrophilic fractions, 0.024–0.236 μmol Fe(II)/g FW for the insoluble-bound fractions, 1.289–11.767 μmol Fe(II)/g FW for total, respectively (Figure 1). The statistical analysis results revealed significant differences among lipophilic, hydrophilic, and insoluble-bound fractions (Table 1). The FRAP values of the 3 fractions were in a decreasing order of lipophilic fraction > hydrophilic fraction > insoluble-bound fraction. The results indicated that most of antioxidants in grape pulps responsible for reducing oxidants were distributed in lipophilic fractions, followed by hydrophilic fractions, with insoluble-bound fractions the least. The grapes with the largest total FRAP values were Pearl Black Grape (Xinjiang, 11.767 μmol Fe(II)/g FW) > Seedless Red Grape (California, 7.880 μmol Fe(II)/g FW) > Summer Black Grape (Shaanxi, 7.830 μmol Fe(II)/g FW) > Pearl Green Grape (Xinjiang, 7.346 μmol Fe(II)/g FW) > Black Grape (Yunnan, 7.267 μmol Fe(II)/g FW) in a decreasing order.

**Figure 2:**
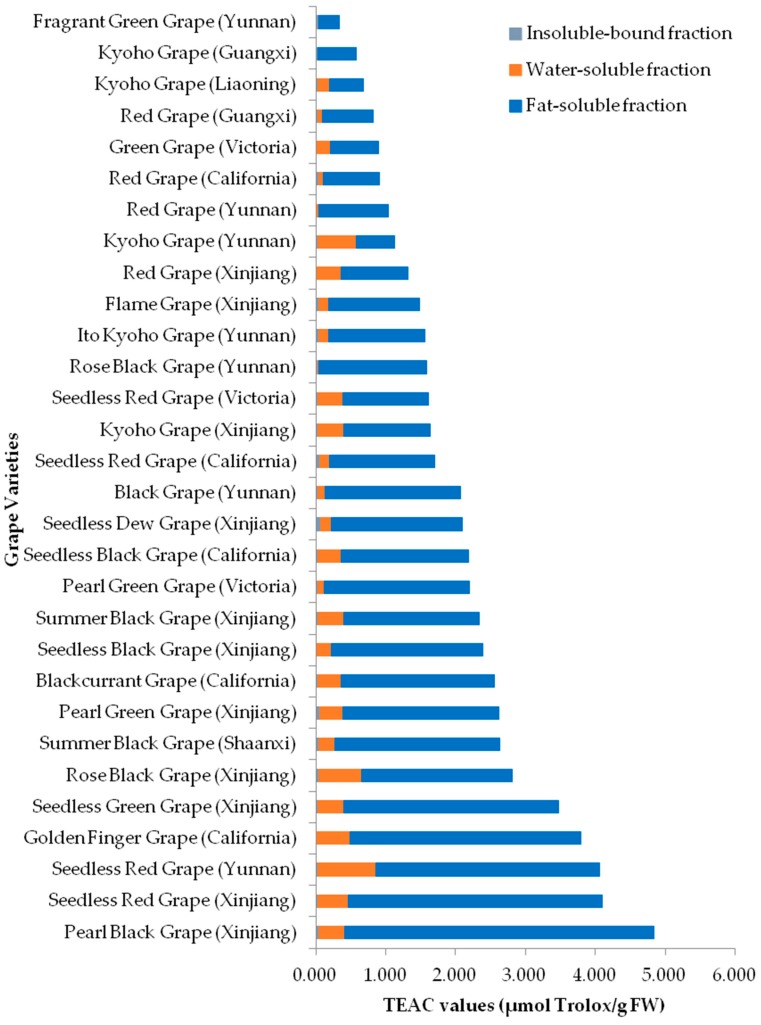
TEAC values of 30 grape pulps.

The TEAC values were in the range of 0.294–4.425 μmol Trolox/g FW for lipophilic fractions, 0.001–0.833 μmol Trolox/g FW for hydrophilic fractions, 0.007–0.064 μmol Trolox/g FW for insoluble-bound fractions, and 0.339–4.839 μmol Trolox/g FW for total, respectively (Figure 2). The TEAC values of 3 fractions were lipophilic fraction > hydrophilic fraction ≈ insoluble-bound fraction (Table 2), indicating that the antioxidants in grape pulps responsible for scavenging free radicals were mostly distributed in lipophilic fractions, followed by hydrophilic and insoluble-bound fractions. The grapes with top-five total TEAC values were Pearl Black Grape (Xinjiang, 4.839 μmol Trolox/g FW) > Seedless Red Grape (Xinjiang, 4.100 μmol Trolox/g FW) > Seedless Red Grape (Yunnan, 4.061 μmol Trolox/g FW) > Golden Finger Grape (California, 3.794 μmol Trolox/g FW) > Seedless Green Grape (Xinjiang, 3.478 μmol Trolox/g FW) in a decreasing order.

The results were consistent with a previous study, in which the antioxidant capacities of 62 fruits were tested [8]. The FRAP values of 4 tested grapes ranged from 1.73 to 10.12 μmol Fe(II)/g wet weight, while TEAC values ranged from 1.23 to 3.95 μmol Trolox/g wet weight. The results of another study [24] showed that FRAP values of 56 wild fruits ranged from 0.67 to 143 μmol Fe(II)/g wet weight, while TEAC values varied from 0.37 to 184 μmol Trolox/g wet weight. The antioxidant capacities of some grape pulps were lower than those of several wild fruits, but grapes are still a better source of antioxidants than wild fruits, as the edibility and toxicity of wild fruits are uncertain.

**Figure 3:**
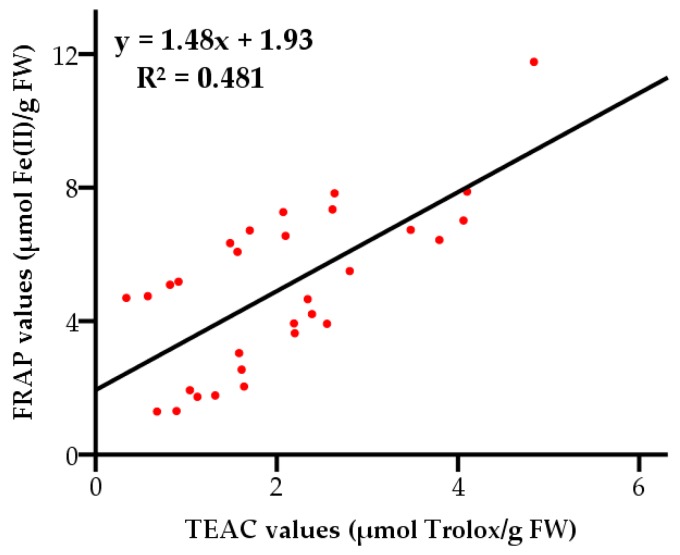
Correlation between total antioxidant activities measured by the FRAP and TEAC assays.

The FRAP and TEAC values showed a moderate positive linear correlation (*R*^2^ = 0.481, with a level of significance of 95%) (Figure 3). The results indicated that the compounds responsible for reducing oxidants were not completely consistent with those scavenging free radicals in grape pulps. It can also be concluded that the antioxidant capacities of grape pulps could not be characterized by a single assay of FRAP or TEAC.

### 2.2. Total Phenolic Contents and Total Flavonoid Contents of Grape Pulps

**Figure 4:**
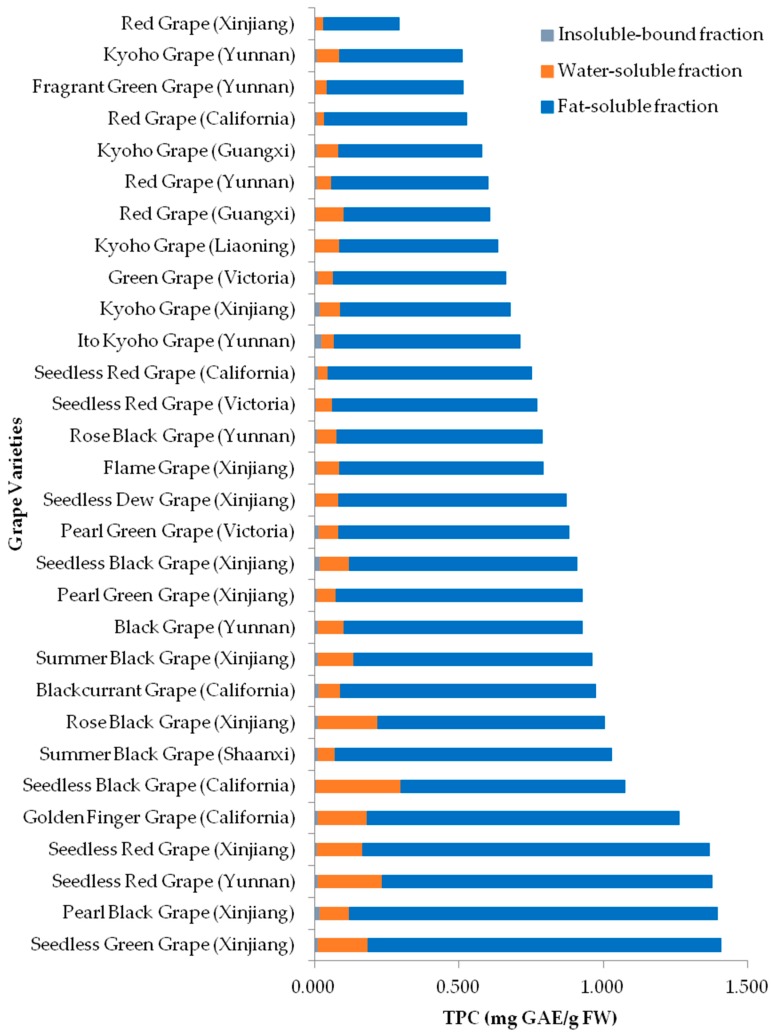
Total phenolic contents (TPC) of 30 grape pulps.

The TPC values ranged from 0.262 to 1.277 milligram gallic acid equivalents (mg GAE)/g FW for the lipophilic fractions, 0.026 to 0.292 mg GAE/g FW for the hydrophilic fractions, 0.004 to 0.026 mg GAE/g FW for the insoluble-bound fractions, and 0.294 to 1.407 mg GAE/g FW for total, respectively (Figure 4). The results of TPC values were consistent with previous studies [25,26]. A study showed the TPC values were 642 ± 9.2 and 1028 ± 14.9 mg GAE/kg fresh material for two grape varieties [25], while in another study, the TPC of pulps from 8 Muscadine grape cultivars were in the range of 0.3–1.2 mg GAE/g FW [26]. The variability of TPC values could be attributed to the genetic and environmental factors of the growing location such as climate, soil composition, temperature, and ripening stage [27]. Another study also suggested that significant varietal differences were observed in phenolic contents among table grape cultivars [18]. The TPC values were in a decreasing order: lipophilic fraction > hydrophilic fraction > insoluble-bound fraction (Table 1). These results might be due to the different polarities of solvents that were used to extract phytochemicals. The weak polarity of tetrahydrofuran made the grape cell membranes easy to dissolve and more permeable; as such higher concentrations of polyphenols were released into the solvents [28]. The grapes with the highest-five total TPC values were Seedless Green Grape (Xinjiang, 1.407 mg GAE/g FW) > Pearl Black Grape (Xinjiang, 1.396 mg GAE/g FW) > Seedless Red Grape (Yunnan, 1.377 mg GAE/g FW) > Seedless Red Grape (Xinjiang, 1.367 mg GAE/g FW) > Golden Finger Grape (California, 1.264 mg GAE/g FW) in a decreasing order.

**Figure 5:**
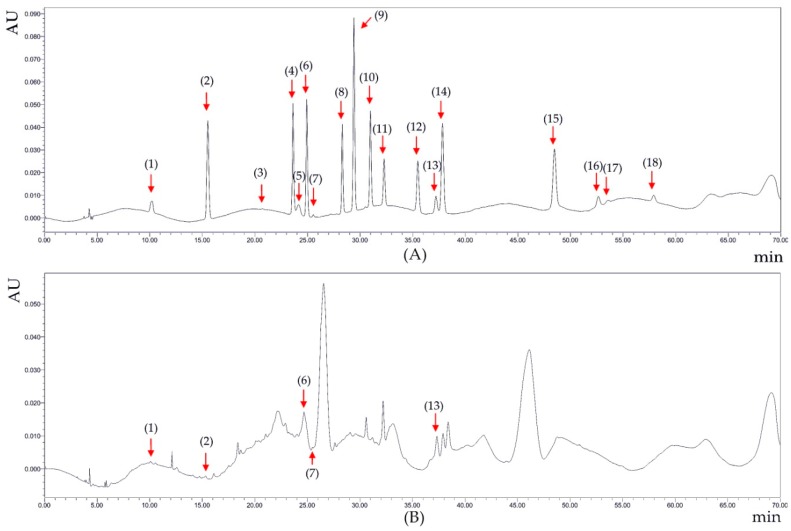
Chromatograms of the standard compounds (**A**) and grape pulp (**B**) under 276 nm. The numbers in brackets refer to the compounds: gallic acid (1); protocatechuic acid (2); gallo catechin (3); chlorogenic acid (4); cyanidin-3-glucoside (5); caffeic acid (6); epicatechin (7); catechin gallate (8); p-coumaric acid (9); ferulaic acid (10); melatonin (11); 2-hydroxycinnamic acid (12); rutin (13); resveratrol (14); daidzein (15); equol (16); quercetin (17); genistein (18).

The main phenolic compounds in grape pulps were tentatively identified according to the retention time and spectra according to the literature [29], and then standard compounds were used to verify the phenolic compounds (Figure 5). The contents of the phenolic compounds were calculated by peak area. Caffeic acid, catechin gallate, epicatechin, gallic acid, protocatechuic acid and rutin were the most widely detected phenolic compounds in the 30 grape pulps (Table 2). The highest contents of caffeic acid, catechin gallate, epicatechin, gallic acid, protocatechuic acid and rutin were found in Green Grape (Victoria, 2.115 μg/g FW), Seedless Red Grape (California, 0.355 μg/g FW), Black Grape (Yunnan, 2.464 μg/g FW), Pearl Black Grape (Xinjiang, 2.262 μg/g FW), Seedless Green Grape (Xinjiang, 1.501 μg/g FW), and Seedless Green Grape (Xinjiang, 8.074 μg/g FW), respectively. That is, the phenolic compositions of grapes might be influenced by the genetic variability and original location. The differences of phenolic compounds among genotypes of *Oenocarpus distichus* Mart. Fruits were also reported in a previous study [30].

Some grape antioxidants have been tested for their effects against a variety of diseases [17]. Among antioxidants in grape pulps, polyphenols were the mostly evaluated regarding preventive effects on diseases like cardiovascular disease [14] and cancer [13]. It can be inferred that grape pulps with effective antioxidant activities and high phenolic contents could be good sources of natural antioxidants to prevent several diseases induced by oxidative stress, and further in vivo investigations need to be conducted with active compounds in grapes.

**Figure 6:**
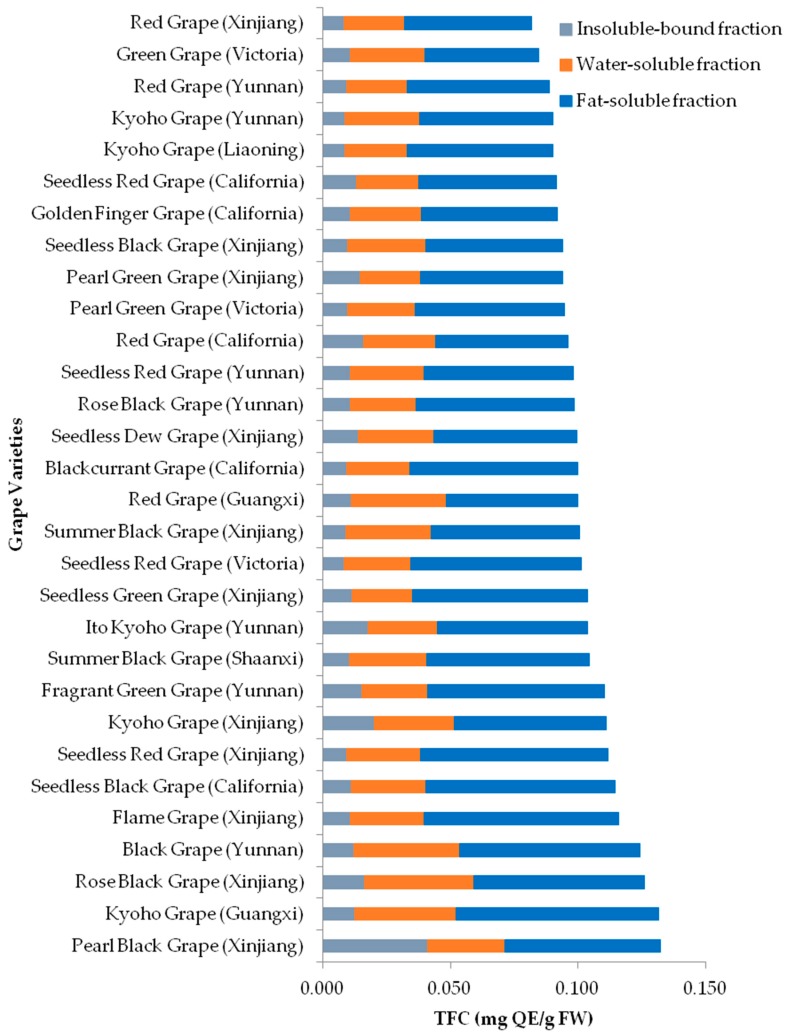
Total flavonoid contents (TFC) of 30 grape pulps.

The TFC values of the 30 grape pulps ranged from 0.044 to 0.079 milligram quercetin equivalents (mg QE)/g FW for the lipophilic fractions, 0.024 to 0.043 mg QE/g FW for the hydrophilic fractions, 0.008 to 0.041 mg QE/g FW for the insoluble-bound fractions, and 0.082 to 0.132 mg QE/g FW for total, respectively (Figure 6). The TFC values of the 3 fractions were lipophilic fraction > hydrophilic fraction > insoluble-bound fraction (Table 1). The grapes with total TFC values were in the order of Pearl Black Grape (Xinjiang, 0.132 mg QE/g FW) > Kyoho Grape (Guangxi, 0.131 mg QE/g FW) > Rose Black Grape (Xinjiang, 0.126 mg QE/g FW) > Black Grape (Yunnan, 0.124 mg QE/g FW) > Flame Grape (Xinjiang, 0.116 mg QE/g FW).

**Figure 7:**
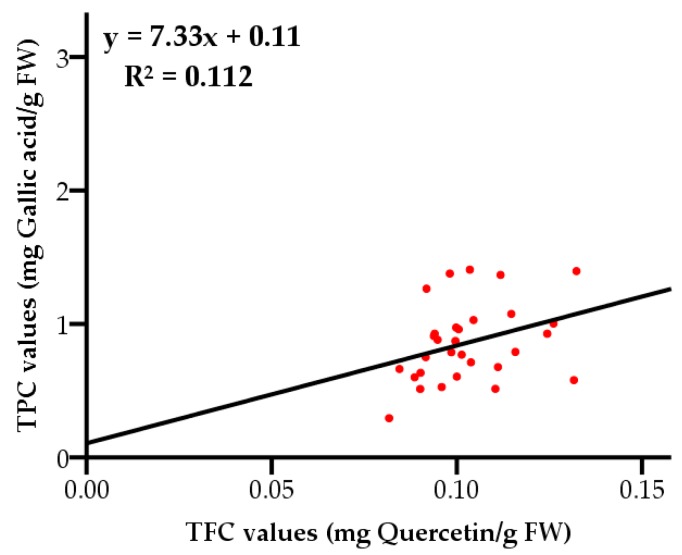
Correlation between total phenolic contents and total flavonoid contents.

The total phenolic contents and total flavonoid contents showed a weak correlation (*R*^2^ = 0.112, with a level of significance of 95%) as displayed in Figure 7, indicating flavonoids were not the main phenolic compounds in grape pulps. The results were different from a previous study, in which the TFC values of three wine grapes were significantly correlated with TPC values [31]. The inconsistency might be a consequence of geographical location and varietal differences.

### 2.3. Correlation between Antioxidant Capacities and Total Phenolic Contents

**Figure 8:**
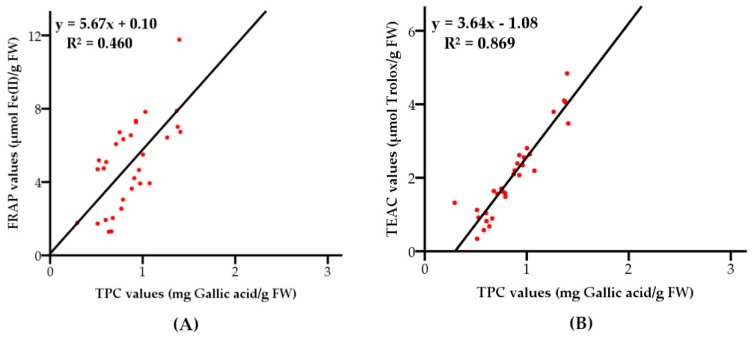
Correlations between FRAP values (**A**), TEAC values (**B**) and total phenolic contents of the 30 grape varieties.

The correlation analysis demonstrated that the level of antioxidant capacities were dependent on total phenolic contents. The correlation coefficient (*R*^2^) between FRAP values and total phenolic contents of the grape pulps was 0.460, with a level of significance of 95%, as shown in Figure 8A. The results indicated that phenolic compounds in grape pulps were moderately related to the capacities of reducing oxidants, which might because of antioxidant vitamins and other antioxidant phytochemicals, and/or synergism among them and polyphenols contributing to FRAP values [32]. The antioxidant capacities investigated by TEAC assay showed highly positive correlation (*R*^2^ = 0.869, with a level of significance of 95%) with total phenolic contents as shown in Figure 8B, indicating phenolic compounds could be the main components responsible for scavenging free radicals. In a previous study, Chichá (*Sterculia striata*) nuts exhibited strong correlations between total phenolic content and antioxidant activities determined by TEAC and FRAP assays [33]. However, no correlation between the TEAC and TPC values of different genotypes of cranberry was found in another study [34].

### 2.4. Correlation between Antioxidant Capacities and Total Phenolic Contents

**Figure 9:**
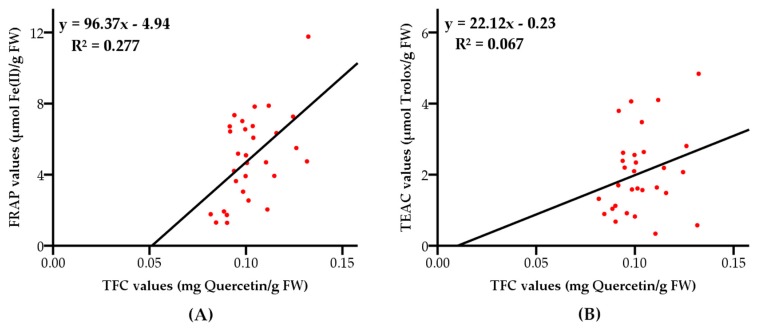
Correlations between FRAP values (**A**), TEAC values (**B**) and total flavonoid contents of the 30 grape varieties.

The correlation coefficient between FRAP, TEAC values and total flavonoid contents were 0.277 (with a level of significance of 95%) and 0.067 (with a level of significance of 90%), as shown in Figure 9, respectively. The results indicated that the reducing oxidants activities exerted by grape pulps rarely depended on flavonoids, and the scavenging free radical activities were hardly dependent on flavonoids.

## 3. Materials and Methods 

### 3.1. Chemicals and Materials

The Folin-Ciocalteu’s phenol reagent, 6-hydroxy-2,5,7,8-tetramethylchromane-2-carboxylic acid (Trolox), 2,4,6-tri(2-pyridyl)-*s*-triazine (TPTZ), 2,2′-azinobis(3-ethylbenothiazoline-6-sulphonic acid) diammonium salt (ABTS), and the standard compounds (gallic acid, protocatechuic acid, gallo catechin, chlorogenic acid, cyanidin-3-glucoside, caffeic acid, epicatechin, catechin gallate, p-coumaric acid, ferulaic acid, melatonin, 2-hydroxycinnamic acid, rutin, resveratrol, daidzein, equol, quercetin and genistein) were obtained from Sigma-Aldrich (St. Louis, MO, USA). Acetic acid, hydrochloric acid, ethanol, *n*-hexane, potassium persulfate, iron(III) chloride hexahydrate, iron(II) sulfate heptahydrate, sodium acetate, sodium carbonate, aluminum chloride, ethylenediaminetetraacetic acid, ascorbic acid, and potassium acetate were of analytical grade and obtained from Damao Chemical Factory (Tianjin, China). Tetrahydrofuran, methanol, diethyl ether and ethyl acetate were of analytical grade and obtained from Kermel Chemical Factory (Tianjin, China). Formic acid and methanol were of chromatographic grade and obtained from Kermel Chemical Factory (Tianjin, China). All the water used in the experiment was double distilled water.

The 30 grapes are commonly consumed varieties in China, and were purchased from local markets in Guangzhou, China. The names and original places of the 30 grape varieties are shown in Table 3.

### 3.2. Sample Extraction

The samples were extracted according to the literature [35,36] with slight modifications. The fresh grapes were cleaned with double distilled water and dried at room temperature, and then the pulps were separated. Immediately, the grape pulps were ground into slurry using a juicer. Tetrahydrofuran (10 mL) was added into accurately-weighted 2.000 g of the slurry, and the mixture was put in a shaking water bath at 30 °C for 30 min. Then, the mixture was centrifuged at 4200× *g* for 10 min. The extraction process was repeated twice, and the supernatants were collected together as the lipophilic fraction.

The residue was mixed with 10 mL acidified methanol (methanol: acetic acid: water = 50:3.7:46.3, *v*/*v*/*v*), and put in a shaking water bath at 30 °C for 30 min, then the mixture was centrifuged at 4200× *g* for 10 min. The residue was extracted twice, and the supernatants were collected as the hydrophilic fraction.

The residue was then hydrolyzed by 5 mL mixture containing 2 mol/L sodium hydroxide, 10 mmol/L ethylenediaminetetraacetic acid, and 1% ascorbic acid, in a shaking water bath at 37 °C for 30 min. The pH of the mixture was adjusted to 2.0 by 6 mol/L hydrochloric acid. The fatty acids generated from hydrolysis were removed twice, by adding 5 mL *n*-hexane, centrifuging (4200× *g*, 10 min), and discarding the organic fraction. The remainder was extracted twice with 5 mL mixture of diethyl ether and ethyl acetate (1:1, *v*/*v*), then the organic fractions were dried under nitrogen and dissolved in 5 mL ethanol as the insoluble-bound fraction. All the extracts were saved at −20 °C.

### 3.3. Determination of FRAP

The FRAP was measured according to the literature [37] with minor modifications. FRAP reagent, a mixture of sodium acetate-acetic acid buffer (300 mmol/L), TPTZ (10 mmol/L) and ferric chloride solution (20 mmol/L) at a volume ratio of 10:1:1, was freshly prepared and put in a water bath at 37 °C before use. One hundred microliters of appropriately diluted samples were mixed with 3 mL FRAP reagent. After incubation for 4 min, the absorbance of the mixture at 593 nm was determined. The results were expressed as μmol Fe(II)/g FW of grape pulps.

### 3.4. Determination of ABTS Free Radical Scavenging Activity

The ABTS free radical scavenging activity was measured by the TEAC assay according to the literature [38,39] with minor modifications. The ABTS^•+^ stock solution was prepared by mixing ABTS (7 mmol/L) solution and potassium persulfate (2.45 mmol/L) in a volume ratio of 1:1, and then incubated in dark at room temperature for at least 16 h and stored less than 2 d before use. The ABTS^•+^ stock solution was diluted with ethanol to an absorbance of 0.710 ± 0.050 at 734 nm. One hundred microliters of appropriately diluted sample was mixed with 3.8 mL ABTS reagent at room temperature, and the absorbance of the mixture at 734 nm was determined after 6 min. The results were expressed as μmol Trolox/g FW of grape pulps.

### 3.5. Determination of TPC

TPC was tested according to the literature [40,41] with minor modifications. Five hundred microliters of appropriately diluted sample was mixed with 2.5 mL 0.2 mol/L Folin-Ciocalteu reagent. Two milliliters saturated sodium carbonate solution (approximately 75 g/L) was added to the mixture after 4 min, followed by 2 h of reaction at room temperature. The absorbance of the mixture was measured at 760 nm. The results were expressed as mg GAE/g FW of grape pulps.

### 3.6. Determination of TFC

TFC was tested according to the literature [42,43] with minor modifications. One point five millilitres ethanol (95%, *v*/*v*), 0.1 mL aluminum chloride (10%, *w*/*v*), 0.1 mL potassium acetate (1 mol/L), and 2.8 mL double distilled water were orderly added into 500 μL of the appropriately diluted sample. After incubation for 30 min at room temperature, the absorbance of the mixture at 415 nm was tested. The results were expressed as mg QE/g FW of grape pulps.

### 3.7. High Performance Liquid Chromatography (HPLC) Analysis

The 3 fractions from grape pulps were mixed and concentrated for HPLC analysis. The phenolic ingredients in 30 grape pulps were analyzed by HPLC-PDAD (photo-diode array detector) according to the method described by Cai et al. [44] with minor modifications. The HPLC system was installed with a Waters (Milford, MA, USA) 1525 binary HPLC pump separation module with an auto-injector and employed a Waters 2996 PDAD. An Agilent Zorbax Extend-C18 column (250 × 4.6 mm, 5 μm) (Santa Clara, CA, USA) was used. Separation was performed at 40 °C with a gradient elution solution A (formic acid solution, 0.1%, *v*/*v*), and solution B (methanol), which were delivered at a flow rate of 0.8 mL/min according to the procedure: 0 min, 95% (A); 15 min, 80% (A); 20 min, 70% (A); 25 min, 63% (A); 40 min, 60% (A); 60 min, 50% (A); 65 min, 50% (A); 65.1 min, 95% (A); and 70 min, 95% (A). The spectra were recorded between 200 and 600 nm for peak characterization. Phenolic compounds were quantified by the peak area of the maximum absorption wavelength.

### 3.8. Statistical Analysis

All the experiments were conducted three times, and the results were expressed as mean ± SD (standard deviation). Statistical analysis was performed using SPSS 22.0 and Excel 2007, and the statistical differences were considered to be significant at the level of *p* < 0.05. 

## 4. Conclusions

The antioxidant capacities, total phenolic contents, and total flavonoid contents of the lipophilic, hydrophilic, and insoluble-bound fractions of 30 grape pulps have been studied systematically. The total FRAP values of 30 grape pulps ranged from 1.289 to 11.767 μmol Fe(II)/g FW, while the total TEAC values varied from 0.339 to 4.839 μmol Trolox/g FW. The total TPC and TFC values of 30 grape pulps ranged from 0.294 to 1.407 mg GAE/g FW and from 0.082 to 0.132 mg QE/g FW, respectively. For 4 parameters, significant differences were observed among 3 fractions, and the values were in such the order of lipophilic fraction > hydrophilic fraction ≥ insoluble-bound fraction. The high correlation between TEAC and TPC, and moderate correlation between FRAP and TPC indicated that phenolic compounds in grape pulps could be the main components responsible for scavenging free radicals, while reducing oxidants powers were not mainly exerted by polyphenols. The weak correlation between FRAP, TEAC and TFC indicated that flavonoids in grape pulps contributed rarely to reducing oxidants activities, and hardly to scavenging free radical activities. Furthermore, caffeic acid, catechin gallate, epicatechin, gallic acid, protocatechuic acid, and rutin were the most widely detectable phenolic compounds in the 30 grape pulps. Finally, several grape varieties with strong antioxidant activities, such as Pearl Black Grape (Xinjiang), Summer Black Grape (Shaanxi), Pearl Green Grape (Xinjiang), Seedless Green Grape (Xinjiang), and Seedless Red Grape (Yunnan), might be potential sources of natural antioxidants, which could be consumed more to prevent some diseases induced by oxidative stress, and could be also cultivated more, leading to widespread health benefits.

## Figures and Tables

**Table 1:** Comparison of the antioxidant activities, TPC and TFC of lipophilic, hydrophilic, and insoluble-bound fractions.

Parameter	Fraction	Mean ± SD	*p*
FRAP values	lipophilic	3.525 ± 1.809 ^a^	<0.001
	hydrophilic	1.393 ± 0.702 ^b^	
	insoluble-bound	0.054 ± 0.039 ^c^	
TEAC values	lipophilic	1.753 ± 1.020 ^a^	<0.001
	hydrophilic	0.263 ± 0.198 ^b^	
	insoluble-bound	0.033 ± 0.013 ^b^	
TPC values	lipophilic	0.753 ± 0.251 ^a^	<0.001
	hydrophilic	0.095 ± 0.063 ^b^	
	insoluble-bound	0.011 ± 0.005 ^c^	
TFC values	lipophilic	0.061 ± 0.009 ^a^	<0.001
	hydrophilic	0.029 ± 0.005 ^b^	
	insoluble-bound	0.013 ± 0.006 ^c^	

^a,b,c^ Different letters within the same parameter indicate a significant difference at *p* < 0.01.

**Table 2:** Main phenolic compounds and their contents in pulps of 30 grape varieties.

Name	Original Place	Phenolic	Content (mean ± SD, μg/g FW)
Black Grape	Yunnan, China	caffeic acid	0.559 ± 0.008
		epicatechin	2.464 ± 0.047
		p-coumaric acid	0.582 ± 0.055
Blackcurrant Grape	California, America	gallic acid	0.363 ± 0.009
		epicatechin	1.237 ± 0.058
		rutin	2.244 ± 0.074
Flame Grape	Xinjiang, China	gallic acid	1.725 ± 0.019
		caffeic acid	0.956 ± 0.017
		epicatechin	1.576 ± 0.013
		p-coumaric acid	0.642 ± 0.006
		rutin	3.067 ± 0.045
Fragrant Green Grape	Yunnan, China	caffeic acid	0.847 ± 0.007
Golden Finger Grape	California, America	gallic acid	0.270 ± 0.004
		caffeic acid	0.590 ± 0.016
		ferulic acid	0.135 ± 0.012
		rutin	5.263 ± 0.074
		catechin gallate	0.344 ± 0.013
Green Grape	Victoria, Australia	protocatechuic acid	0.405 ± 0.001
		caffeic acid	2.115 ± 0.026
Ito Kyoho Grape	Yunnan, China	protocatechuic acid	0.452 ± 0.017
		caffeic acid	0.650 ± 0.019
Kyoho Grape	Guangxi, China	caffeic acid	0.962 ± 0.024
Kyoho Grape	Liaoning, China	catechin gallate	0.185 ± 0.015
Kyoho Grape	Xinjiang, China	caffeic acid	1.157 ± 0.046
		catechin gallate	0.219 ± 0.004
Kyoho Grape	Yunnan, China	epicatechin	0.654 ± 0.031
Pearl Black Grape	Xinjiang, China	gallic acid	2.262 ± 0.051
		caffeic acid	0.688 ± 0.045
		epicatechin	0.976 ± 0.025
Pearl Green Grape	Xinjiang, China	gallic acid	1.430 ± 0.074
		protocatechuic acid	0.210 ± 0.005
		caffeic acid	0.415 ± 0.004
		epicatechin	0.630 ± 0.013
		rutin	3.503 ± 0.058
Pearl Green Grape	Victoria, Australia	gallic acid	0.274 ± 0.001
		caffeic acid	0.910 ± 0.013
Red Grape	California, America	protocatechuic acid	0.338 ± 0.033
Red Grape	Guangxi, China	protocatechuic acid	0.321 ± 0.007
Red Grape	Xinjiang, China	caffeic acid	0.848 ± 0.039
Red Grape	Yunnan, China	gallic acid	0.219 ± 0.002
		caffeic acid	0.301 ± 0.020
Rose Black Grape	Xinjiang, China	caffeic acid	0.829 ± 0.055
Rose Black Grape	Yunnan, China	protocatechuic acid	0.240 ± 0.001
		epicatechin	1.439 ± 0.027
		rutin	1.369 ± 0.018
Seedless Black Grape	California, America	epicatechin	1.586 ± 0.091
Seedless Black Grape	Xinjiang, China	gallic acid	0.313 ± 0.007
		caffeic acid	1.285 ± 0.069
		epicatechin	1.338 ± 0.023
		ferulic acid	0.613 ± 0.030
		rutin	1.822 ± 0.023
Seedless Dew Grape	Xinjiang, China	protocatechuic acid	0.157 ± 0.002
		caffeic acid	0.457 ± 0.037
		epicatechin	1.235 ± 0.011
		rutin	1.267 ± 0.026
Seedless Green Grape	Xinjiang, China	protocatechuic acid	1.501 ± 0.035
		caffeic acid	0.798 ± 0.042
		epicatechin	0.762 ± 0.055
		rutin	8.074 ± 0.094
Seedless Red Grape	California, America	protocatechuic acid	0.143 ± 0.008
		caffeic acid	1.048 ± 0.010
		rutin	2.277 ± 0.053
		catechin gallate	0.355 ± 0.023
Seedless Red Grape	Victoria, Australia	gallic acid	0.262 ±0.012
		protocatechuic acid	0.371 ± 0.023
		caffeic acid	0.770 ± 0.063
		epicatechin	1.053 ± 0.065
Seedless Red Grape	Xinjiang, China	caffeic acid	0.879 ± 0.036
Seedless Red Grape	Yunnan, China	gallic acid	0.413 ± 0.020
		epicatechin	0.649 ± 0.014
		rutin	1.950 ± 0.062
Summer Black Grape	Shaanxi, China	gallic acid	0.658 ± 0.053
		caffeic acid	1.488 ± 0.047
		epicatechin	1.431 ± 0.075
		rutin	3.770 ± 0.004
Summer Black Grape	Xinjiang, China	protocatechuic acid	0.353 ± 0.033
		caffeic acid	1.052 ± 0.011
		epicatechin	2.263 ± 0.095

**Table 3:** Names and original places of the 30 grape varieties.

No.	Name	Original Places
1	Black Grape	Yunnan, China
2	Blackcurrant Grape	California, America
3	Flame Grape	Xinjiang, China
4	Fragrant Green Grape	Yunnan, China
5	Golden Finger Grape	California, America
6	Green Grape	Victoria, Australia
7	Ito Kyoho Grape	Yunnan, China
8	Kyoho Grape	Guangxi, China
9	Kyoho Grape	Liaoning, China
10	Kyoho Grape	Xinjiang, China
11	Kyoho Grape	Yunnan, China
12	Pearl Black Grape	Xinjiang, China
13	Pearl Green Grape	Xinjiang, China
14	Pearl Green Grape	Victoria, Australia
15	Red Grape	California, America
16	Red Grape	Guangxi, China
17	Red Grape	Xinjiang, China
18	Red Grape	Yunnan, China
19	Rose Black Grape	Xinjiang, China
20	Rose Black Grape	Yunnan, China
21	Seedless Black Grape	California, America
22	Seedless Black Grape	Xinjiang, China
23	Seedless Dew Grape	Xinjiang, China
24	Seedless Green Grape	Xinjiang, China
25	Seedless Red Grape	California, America
26	Seedless Red Grape	Victoria, Australia
27	Seedless Red Grape	Xinjiang, China
28	Seedless Red Grape	Yunnan, China
29	Summer Black Grape	Shaanxi, China
30	Summer Black Grape	Xinjiang, China
